# Exploring the mechanism of traditional Chinese medicine in regulating gut-derived 5-HT for osteoporosis treatment

**DOI:** 10.3389/fendo.2023.1234683

**Published:** 2023-10-17

**Authors:** Kai Sun, Yincang Wang, Jiazhe Du, Yujie Wang, Bo Liu, Xiaodong Li, Xiaofeng Zhang, Xilin Xu

**Affiliations:** ^1^ The First Department of Orthopedics and Traumatology, The First Affiliated Hospital of Heilongjiang, University of Chinese Medicine, Harbin, Heilongjiang, China; ^2^ Heilongjiang University of Chinese Medicine, Harbin, Heilongjiang, China; ^3^ The First Department of Orthopedics and Traumatology, The Third Affiliated Hospital of Heilongjiang University of Chinese Medicine, Harbin, Heilongjiang, China; ^4^ Teaching and Research Section of Orthopedics and Traumatology, Heilongjiang University of Chinese Medicine, Harbin, Heilongjiang, China

**Keywords:** osteoporosis, gut-derived 5-hydroxytryptamine, low density lipoprotein receptor-related protein 5, 5-HT receptor, intestinal microbioms

## Abstract

Osteoporosis is a systemic bone disease characterized by an imbalance in the relationship between osteoblasts, osteocytes, and osteoclasts. This imbalance in bone metabolism results in the destruction of the bone’s microstructure and an increase in bone brittleness, thereby increasing the risk of fractures. Osteoporosis has complex causes, one of which is related to the dysregulation of 5-hydroxytryptamine, a neurotransmitter closely associated with bone tissue metabolism. Dysregulation of 5-HT directly or indirectly promotes the occurrence and development of osteoporosis. This paper aims to discuss the regulation of 5-HT by Traditional Chinese Medicine and its impact on bone metabolism, as well as the underlying mechanism of action. The results of this study demonstrate that Traditional Chinese Medicine has the ability to regulate 5-HT, thereby modulating bone metabolism and improving bone loss. These findings provide valuable insights for future osteoporosis treatment.

## 1 Introduction

Osteoporosis (OP) is a systemic skeletal disease characterized by reduced bone mass and degradation of bone tissue microarchitecture, leading to increased bone fragility, decreased bone strength, and high susceptibility to fractures. (1). Bone remodeling is a continuous and intricate process. It involves osteoclasts in bone resorption to maintain bone shape, while osteoblasts primarily participate in bone formation to promote bone deposition. The balance between bone formation and resorption is crucial for maintaining bone tissue health. Disturbances in this balance can cause osteoporosis, osteosclerosis, and fractures (2). The prevalence of osteoporosis has been increasing steadily in recent years due to changes in the world’s demographic structure and the deepening aging trend. This disease has had a significant impact on personal and family life, as well as increasing the burden on society to some extent. Therefore, it is crucial and meaningful to explore treatment options for osteoporosis.

In recent years, the role of 5-HT (5-Hydroxytryptamine, or serotonin) in bone metabolism has received significant attention, given its association with bone metabolism, neurotransmitter presence in bone tissue, and regulation of bone reconstruction via two distinct pathways (3). Although the treatment of osteoporosis with Chinese herbs is characterized by multi-target effects and low toxicity and side effects, a review on the modulation of 5-HT by Chinese herbs for the treatment of osteoporosis has not yet been published. Therefore, this paper aims to present the mechanism of action of Chinese herbs in modulating 5-HT for the treatment of osteoporosis.

## 2 Medication treatment for osteoporosis

Currently, the clinical application of anti-osteoporosis drugs mainly involves medications that inhibit bone resorption and those that promote bone formation. The mainstream medications for osteoporosis include bisphosphonates, estrogen, and parathyroid hormone (4–6). However, there are several issues associated with these drugs. Firstly, these medications have a narrow therapeutic focus and may not provide comprehensive and effective treatment for osteoporosis. Secondly, they often result in side effects such as gastrointestinal discomfort and an increased risk of venous thrombosis and stroke (7, 8). Additionally, certain drugs may elevate the chances of hypercalcemia and bone malignancies (9, 10). Thus, the challenge of effectively treating osteoporosis with safe drugs persists.

Chinese medicines have been found to be effective in treating osteoporosis through various mechanisms. Firstly, they can reduce oxidative stress and promote bone health through their antioxidant effects (11, 12). Secondly, Chinese medicines can regulate the balance of intestinal flora, which is crucial for maintaining bone health (13, 14). Additionally, the regulatory effects of Chinese medicines can also impact the function of the endocrine system, which is considered to be another mechanism by which they treat osteoporosis (15). Clinical practice and animal experiments have further evidenced the effectiveness of Chinese herbal formulas in treating osteoporosis. Additionally, animal experiments have shown that specific Chinese herbs can promote bone regeneration and enhance bone strength (16). Chinese medicine, which has been developed and applied for thousands of years, utilizes natural plant, animal, and mineral-derived raw materials. These materials have multiple targets of action, exhibit remarkable efficacy, and produce fewer toxic side effects. As a result, Chinese medicine offers significant advantages in alleviating the clinical symptoms associated with osteoporosis (17, 18).

## 3 The role and regulatory mechanisms of gut-derived 5-HT in osteoporosis

**Figure 1:**
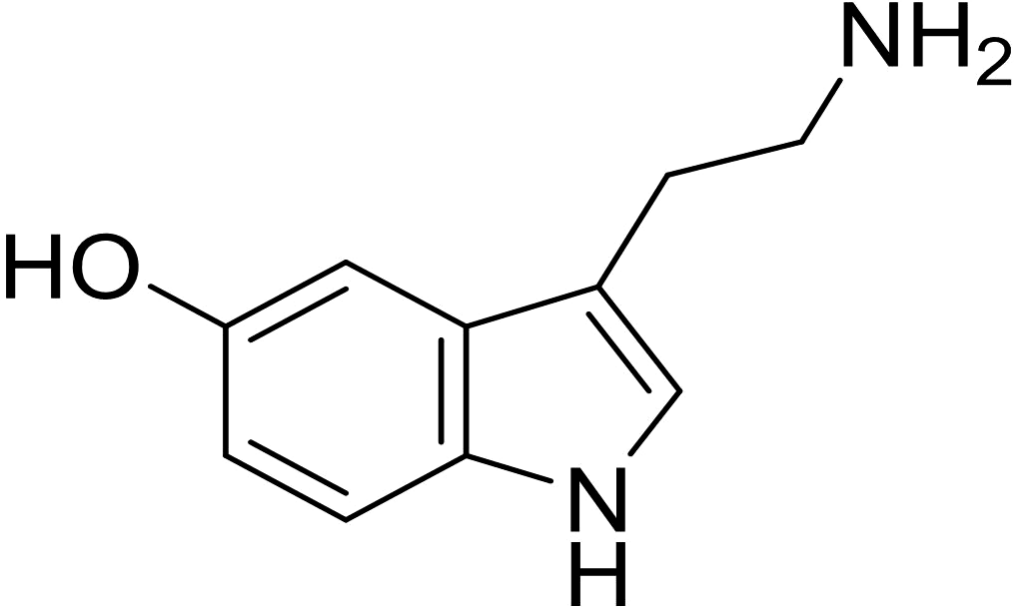
The chemical structure of 5-Hydroxytryptamine.

5-HT, also known as serotonin, is an indoleamine compound consisting of indole and ethylamine, with a molecular formula of C_10_H_12_N_2_O and a molecular weight of 176.2.Tryptophan hydroxylase synthesizes 5-hydroxytryptophan, which is then converted into 5-hydroxytryptamine by 5-hydroxytryptophan dehydroxylase (**Figure 1**) (19, 20). There are two types of 5-HT: central 5-HT and peripheral 5-HT.Central 5-HT, also known as brain-derived 5-HT, is produced by 5-HT neurons in the raphe nucleus of the brainstem. On the other hand, peripheral 5-HT, referred to as gut-derived 5-HT, is produced by duodenal chromaffin cells. The production of central 5-HT is limited by Tph2(tryptophan hydroxylase 2), whereas Tph1(tryptophan hydroxylase 1) limits the production of peripheral 5-HT.Peripheral blood-derived serotonin (5-HT) is unable to cross the blood-brain barrier, indicating that central and peripheral 5-HT are separate systems with different functions (21, 22). Central 5-HT promotes bone formation and suppresses bone resorption, while peripheral 5-HT promotes bone resorption.

In the human body, only 5% of 5-HT is found in the central nervous system, while 90% is in the gastrointestinal system and 5% exists in platelets. Gut-derived 5-HT is produced in EC cells and enters circulation to be taken up by platelets. In tissue damage and inflammation, platelets release 5-HT, which diffuses and binds to various receptors to exert its biological activity (21, 23). 5-HT in bone tissue has multiple sources. It can act as a neurotransmitter, regulated by both sympathetic and hypothalamic involvement(24). Additionally, osteoblasts are capable of producing 5-HT, and the presence of tryptophan hydroxylase-1 mRNA has been observed in both osteoblasts and osteoclast lines (25). A portion of the 5-HT in bone tissue may originate from the bloodstream, as platelets, which store 5-HT, can release it when they come into contact with osteoblasts or are in close proximity to them (26). The role and expression of 5-HT and 5-HT transporter(5-HTT) in bone tissue have been extensively studied. Through release and reuptake of 5- HT, 5-HT and 5-HTT regulate bone metabolism (25–27).

**Figure 2:**
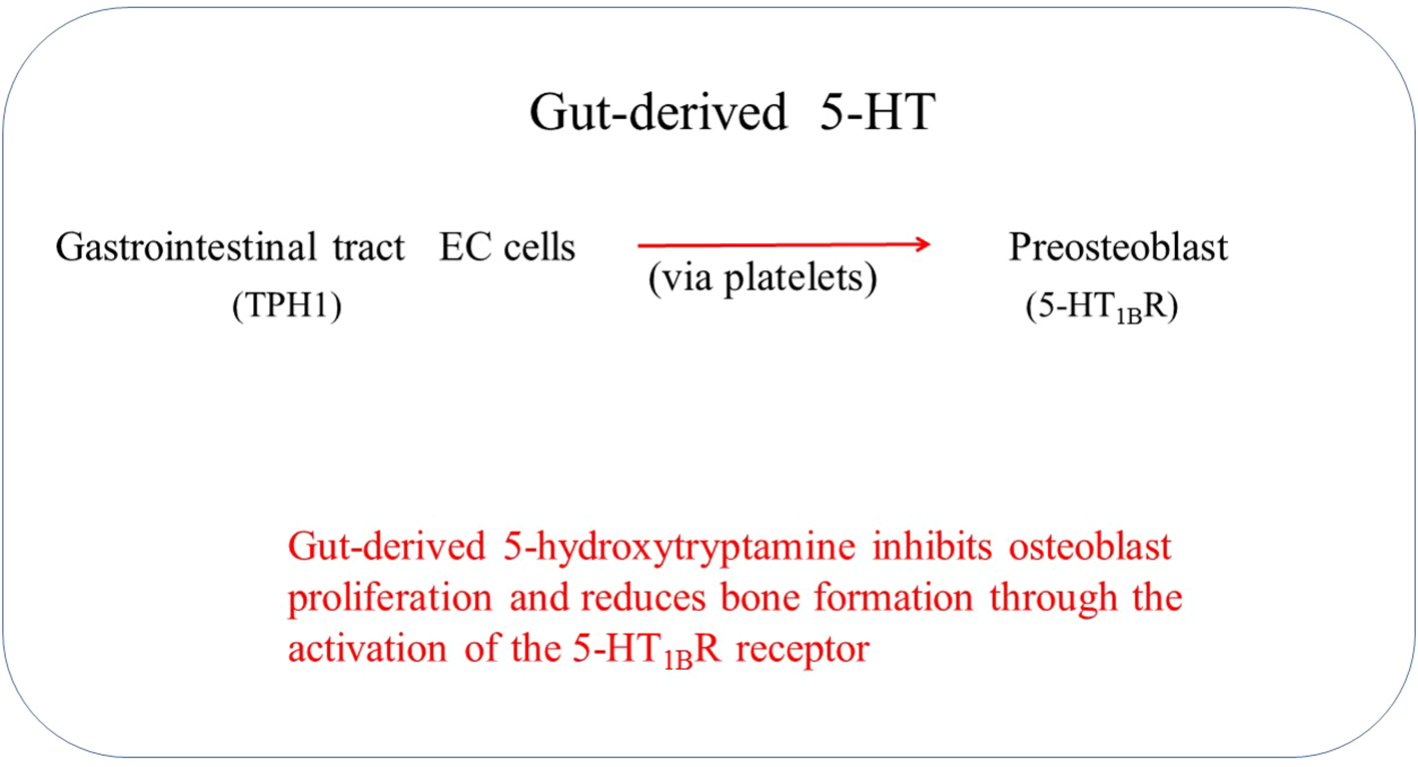
Gut-derived 5-HT regulates the increase in bone mass.

Gut-derived 5-HT is the primary source of skeletal 5-HT; thus, it is suggested that gut-derived 5-HT acts as an endocrine signaling molecule (**Figure 2**) (28). Low-density lipoprotein receptor-related protein 5 (Lrp5) is one of the critical regulatory genes for postnatal bone formation in humans. Lrp5 controls serotonin synthesis by enterochromaffin cells and releases it into the blood circulation (29). Establishing a link between Lrp5 and gut-derived 5-HT suggests a possibility of interaction between gut-derived 5-HT and bone metabolism (30). Lrp5 serves as an anabolic mediator in bone by facilitating Wnt signaling. Alterations in Lrp5, whether through gain or loss, have been linked to high bone mass syndrome and osteoporosis (31–33).

## 4 The research progress of Chinese medicine in the regulation of gut-derived 5-HT for the treatment of osteoporosis

Lignans, being phytoestrogens, are bioactive compounds that possess various biological properties, such as anti-inflammatory, antioxidant, and anti-osteoporotic effects (34, 35). In particular, the lignan-rich fraction obtained from Sambucus Williamsii Ramulus has demonstrated efficacy in the treatment of bone and joint-related disorders by enhancing bone mass, structure, and facilitating beneficial bone remodeling. Sambucus Williamsii Ramulus, a plant known for its medicinal properties, provides a valuable source of lignans that can contribute to the improvement of bone health (36). In a study involving ovariectomized (OVX) rats, it was found that injection of SWRH, a lignan-rich component, significantly inhibited bone loss and improved bone microstructure. This effect was attributed to SWRH’s ability to down-regulate Tph1 mRNA and protein expression, thereby inhibiting serotonin synthesis in RBL-2H3 cells. Furthermore, *in vivo* studies showed that SWRH exerted a protective effect on bones by inhibiting gut-derived 5-HT (37). Another lignan-rich component, SWCA, was found to inhibit the expression of TPH-1 protein in the colon of OVX rats, but not TPH-2 protein in the brain. Additionally, SWCA was shown to reverse the gene and protein expression of FOXO1 and ATF4, which are involved in the serotonin receptor 5HTR1b/CREB/cyclin signaling cascade in the femur of OVX rats. These findings suggest that SWCA indirectly protects against osteoporosis by inhibiting gut-derived 5-HT through the inhibition of intestinal TPH-1 protein and regulation of gut microbiota composition (38).

Arecanut is a type of traditional Chinese medicine that exhibits numerous pharmacological activities, including anti-parasitic, anti-oxidative, anti-inflammatory, analgesic, and digestive effects (39). ACP, a polyphenol derived from Areca catechu seeds, has been found to possess anti-osteoporotic effects by elevating lysozyme expression and maintaining the number of Paneth cells in OVX rats, regulating the intestinal microbiota, and alleviating osteoporosis by inhibiting inflammatory responses (40). Further studies showed that ACP can significantly down-regulate 5HTR1b receptor expression, increase the ratio of OPG/RANKL, Lrp5, BMP2, and β-catenin while promoting expression of metabolites, such as 7-Ketodeoxycholic acid, indole and 15-Deoxy-Δ12,14-prostaglandin, that are closely involved in 5-HT synthesis via Lrp5 and tryptophan metabolism. These results suggest that ACP can modify the Lrp5/TPH1/5-HT signaling pathway, down-regulate 5-HT production, activate the osteoblast Wnt/β-catenin pathway, and improve osteoporosis (41).

Tph1 is a rate-limiting enzyme involved in gut-derived 5-HT biosynthesis and represents a novel target for treating osteoporosis. Studies involving the design and synthesis of a novel ursolic acid derivative, 9a, demonstrated that it inhibits Tph-1 protein and mRNA expression, reduces serotonin content in serum and the intestinal tract without affecting brain serotonin, and improves bone microstructure of OVX rats, all without estrogenic side effects (42). In further studies, ursolic acid derivative 8d significantly inhibited protein and mRNA expression of Tph-1 and prevented bone loss by inhibiting intestinal-derived 5-HT biosynthesis in OVX rats, without affecting brain-derived 5-HT. Moreover, there were no significant estrogenic side effects with high doses of 8d (43).

Bushen zhuanggu granule, a formulation consisting of numerous traditional Chinese medicines, has been developed over many years of clinical practice. Clinical application has shown that Bushen zhuanggu granule exerts a positive therapeutic effect on osteoporosis (44, 45). Further research showed that administration of Bushen Zhuanggu Granules to patients with primary osteoporosis resulted in increased bone mineral density at Ward’s triangle and the left proximal femur, as well as a significant decrease in serum 5-HT levels. This suggests that Bushen Zhuanggu Granules can improve osteoporosis by regulating spleen and kidney function, reducing the synthesis of gut-derived 5-HT, and increasing bone density (46). The characteristics of the included studies are summarized in **Table 1**.

**Table 1:** Traditional Chinese medicine regulates gut-derived 5-HT to treat osteoporosis.

Species	Subjects	Effects and mechanisms	Ref
Lignans (SWRH)	ovariectomized rat	SWR can inhibit the synthesis of 5-hydroxytryptamine and down-regulate the mRNA and protein expression of TPH-1 in RBL-2H3 cells.	(37)
Lignans (SWAC)	ovariectomized rat	SWA treatment reversed the gene and protein expressions of FOXO1 and ATF4.	(38)
arecanut (*Areca catechu* L.) seed polyphenol (ACP)	ovariectomized rat	ACP increases LYZ expression by maintaining the number of Paneth cells and ameliorates osteoporosis by controlling the inflammatory response.	(40)
arecanut (*Areca catechu* L.) seed polyphenol (ACP)	ovariectomized rat	ACP altered the Lrp5/Tph1/5-HT signaling pathway and the down-upregulated 5-HT increased the osteoblast leading to the activation of wnt/β-catenin.	(41)
ursolic acid derivative 9a	ovariectomized rat	9a can inhibit the protein and mRNA expression of Tph-1, and reduce the serotonin content in serum and intestinal tract.	(42)
ursolic acid derivative 8d	ovariectomized rat	8d inhibited protein and mRNA expression of TPH-1 and prevented 5-HT synthesis.	(43)
Bushen Zhuanggu Granules	Primary osteoporosis patients	regulating spleen and kidney function, reducing the synthesis of gut-derived 5-HT, and increasing bone density.	(46)

## 5 The mechanism of traditional Chinese medicine in regulating gut-derived 5-HT for the treatment of osteoporosis

### 5.1 Inhibition of gut-derived 5-HT synthesis

The analysis of bone RNA in wild-type and Lrp5-deficient mice showed that Lrp5 inhibits the expression of Tph1, leading to reduced 5-HT synthesis and indirectly promoting bone formation.This inhibition occurs through the binding of serotonin to the HT1_B_R receptor,which subsequently inhibits the expression of CREB and impacts osteoblast proliferation (28). Gut-derived 5-HT has been demonstrated to act on 5-HT_1B_R, reducing the expression of osteoblast cyclin genes (CyclinD1, D2, and E1) by inhibiting cAMP production and PKA-mediated CREB phosphorylation.It mediates osteoblast proliferation through the HT1_B_R/PKA/CREB/cyclins signaling cascade, regulating osteoblast number and bone mass (47). These results highlight the importance of gut-derived serotonin and its interaction with specific receptors in regulating bone mass.

ACP can significantly downregulate the expression of the 5HT1BR receptor and upregulate Lrp5 expression to inhibit the synthesis of 5-HT, thereby regulating bone mass. The administration of Bushen zhuanggu granule has been shown to improve osteoporosis by regulating the functions of the spleen and kidney, reducing the synthesis of enterogenous 5-HT, and lowering its concentration in the serum.

### 5.2 Downregulating TPH-1 to inhibit gut-derived 5-HT synthesis

Tph1, the rate-limiting enzyme in the biosynthesis of intestinal-derived 5-HT, has potential for the treatment of osteoporosis. Inhibiting Tph1 reduces the synthesis of gut-derived 5-HT, which decreases the role of serotonin in osteoblasts, promotes bone formation, and slows the progression of osteoporosis (48). It was discovered that the small molecule inhibitor LP533401 was employed to suppress the activity of Tph1, and the therapeutic impact of Tph1 inhibition on osteoporosis was observed in a mouse model. Specifically, in aging mice, the inhibition of Tph1 improved bone loss resulting from ovariectomy (49).

SWRH inhibits the synthesis of intestinal 5-HT by downregulating the expression of Tph-1 mRNA and protein. Additionally, the derivatives 9a and 8d of ursolic acid can also suppress the protein and mRNA expression of Tph-1, leading to a decrease in serotonin levels in the serum and intestines. These effects contribute to the improvement of osteoporosis symptoms.

### 5.3 Regulation of the balance between the transcription factors FOXO1, ATF4, and CREB

Gut-derived serotonin indirectly regulates bone tissue through various pathways. The Forkhead transcription factor O subtype 1 (FOXO1), Activator of transcription 4 (ATF4), and Cyclic AMP response element-binding protein (CREB) play a crucial role in promoting osteoblast proliferation by balancing each other. The combined action of 5-HT and 5-HT_1B_R regulates the transcriptional activity of FOXO1 in cells. FOXO1 and ATF4 inhibit osteoblast production, while FOXO1 and CREB promote it. They maintain balance when serum 5-HT levels are normal. However, increased serum 5-HT inhibits the binding of FOXO1 to CREB. It also enhances the binding of FOXO1 to ATF4.This imbalance results in decreased bone mass (50).

The lignan-rich fraction from Sambucus williamsii Hance has been shown to reverse the gene and protein expressions of FOXO1 and ATF4 in the femur. FOXO1 and ATF4 are key players in the serotonin receptor 5HTR1b/CREB/cyclins signaling cascade, which is involved in bone remodeling. In ovariectomized rats, the gene and protein expressions of ATF4 and FOXO1 are dysregulated, leading to an imbalance in bone remodeling processes. However, treatment with the lignan-rich fraction (SWCA) restores the expression levels of these proteins to normal. Specifically, SWCA treatment downregulates the gene expression of ATF4 and upregulates the gene expression of FOXO1 in the femur. This modulation of gene expression is accompanied by corresponding changes in protein expression. The lignan-rich fraction helps to restore the balance between ATF4 and FOXO1, which is important for proper bone formation and remodeling. By reversing the dysregulation of FOXO1 and ATF4 in the femur, the lignan-rich fraction from Sambucus williamsii Hance contributes to its bone protective effects and helps maintain bone health.

### 5.4 Modulation of the intestinal microbiome to inhibit gut-derived 5-HT

In recent years, research has revealed that the gut microbiota plays a significant role in bone metabolism through its influence on host metabolism, immune function, and hormone secretion. Gut-derived 5-HT regulates several bodily functions, including intestinal motility, immune response, secretory response, cardiac function, bone development, and platelet aggregation (51–53). Previous studies have discovered that the 5-HT may be produced by the bacteria Corynebacterium, Streptococcus, and E. coli (54). Another study found that the expression of tryptophan hydroxylase 1, the rate-limiting enzyme in the synthesis of 5-HT, is upregulated in the presence of propionic acid and butyric acid produced by intestinal flora. This upregulation results in elevated levels of 5-HT originating from the intestines (55, 56). Chronic high-dose alcohol consumption increases the risk of bone damage and fracture, while also directly impacting gut microbial composition (57, 58). Recent studies have demonstrated that chronic high-dose alcohol consumption in rats leads to osteoporosis and dysregulation of gut microbial composition. Furthermore, elevated levels of gut-derived serotonin were found to be positively correlated with changes in gut microbial composition. *In vitro* results indicate that elevated 5-HT levels inhibit osteoblast proliferation and mineralization, ultimately affecting bone metabolism (59).

The gut microbiota has the ability to regulate the synthesis and metabolism of 5-HT by influencing microbial metabolites. Moreover, the composition of the gut microbiota plays a crucial role in regulating gut microbial metabolites, host metabolism, and bone metabolism, all of which have an impact on the development and progression of osteoporosis. Consequently, the lignan-rich components found in Sambucus williamsii Hance can indirectly inhibit the synthesis of gut-derived 5-HT by modulating the composition of the gut microbiota. This modulatory effect contributes to the maintenance of normal 5-HT levels, thereby positively influencing bone protection.

## 6 Analysis of conflicting views on whether modulation of gut-derived 5-HT can be an effective treatment for osteoporosis

Recently, there has been an increasing interest in studying Lrp5, gut-derived 5-HT, and their associated receptors. This heightened interest can be attributed to several factors, including the potential roles of these associated receptors in diverse physiological processes and their potential as therapeutic targets. However, despite the growing interest, the findings in this area have sparked intense debates and yielded conflicting conclusions.

The examination of bone remodeling and biochemical markers in mice of various ages and genotypes revealed a significant reduction in bone resorption in mice lacking peripheral serotonin. This finding suggests that serotonin plays a role in bone resorption regulation. Furthermore, it was demonstrated that osteoblast precursor cells express TPH1 and synthesize serotonin in the presence of RANKL. This indicates that serotonin production is stimulated by RANKL, a key regulator of osteoblastogenesis. Moreover, serotonin was found to enhance the effect of RANKL on osteoblastogenesis, suggesting a positive feedback loop between serotonin and RANKL in bone remodeling (60). It has been discovered that long-term injections of serotonin in rats led to a significant increase in bone density, positive alteration in bone structure, and a substantial increase in thigh bone strength. This suggests that serotonin may have a positive impact on bone *in vivo* by either reducing bone resorption or enhancing bone deposition (61). The impact of Lrp5 on bone mass was also examined through knockout experiments, yielding contrasting results compared to Yadav’s study. Contrary to Yadav’s findings, these experiments indicate that Lrp5 does not indirectly regulate bone formation through the inhibition of peripheral serotonin synthesis. Instead, activating the HBM Lrp5 allele on osteoblasts leads to increased bone mass, while inactivating Lrp5 on osteocytes results in decreased bone mass. Interestingly, the inactivation of intestinal Lrp5 did not lead to abnormal bone mass (62).

Based on available studies, it is hypothesized that Lrp5 directly regulates osteoblasts via the classical Wnt pathway. Additionally, increasing circulating 5-HT levels may indirectly regulate preosteoblasts. These mechanisms appear plausible and may complement each other in physiological processes. Gut-derived 5-HT may significantly influence bone formation regulation. However, additional studies are required to determine the precise mechanism and extent of its effects. These studies could investigate the interactions between 5-HT and osteoblasts, as well as the specific mechanism of 5-HT’s action in bone formation. Therefore, additional studies are essential to uncover the mechanisms through which 5-HT regulates bone formation and the extent of its effects. In-depth study of the role of 5-HT can enhance our understanding of the physiological process of bone formation and contribute novel ideas and methods for treating related diseases.

The conflicting conclusions stem from variations in experimental design, sample sizes, and methodologies employed in the studies. These discrepancies underscore the necessity for additional research to elucidate the role of Lrp5, gut-derived 5-HT, and their associated receptors, aiming to achieve a consensus in the scientific community.

## 7 Discussion

Despite ongoing controversies surrounding the findings, there has been significant progress made in this field, With this improved understanding of osteoporosis pathogenesis and detailed analysis of 5-HT-related systems, it appears that these systems could serve as promising targets for innovative therapies that may improve OP and impact bone metabolism. Previous studies have shown that brain-derived 5-HT promotes bone formation, whereas gut-derived 5-HT inhibits osteogenesis, presenting a new treatment approach for OP. Moreover, it has been demonstrated that Chinese herbal formulations can improve the function of the hypothalamus-pituitary-ovary axis in OVX rats and normalize the neuroendocrine state, including the positive regulation of brain-derived serotonin (63). This positive regulation of brain-derived serotonin is crucial in addressing OP. Increasing brain-derived 5-HT levels and decreasing gut-derived 5-HT levels effectively counter the effects of OP.

The potential mode of action for natural herbs in treating osteoporosis by modulating gut-derived 5-HT remains largely unexplored. Conducting both *in vitro* and *in vivo* experiments is necessary to comprehensively assess the safety, efficacy, and interaction of these herbs with other therapeutic modalities. This will enable the determination of whether these natural remedies are capable of serving as a viable alternative to primary osteoporosis medications or as a complement to existing therapies. By combining various modalities to assist in the management of osteoporosis, better clinical outcomes can be achieved.

## 8 Conclusions

The regulation of gut-derived 5-HT by natural herbal medicine is crucial for the treatment of osteoporosis. It achieves the treatment of osteoporosis through various pathways, including inhibiting the synthesis of gut-derived 5-HT, downregulating TPH-1 to suppress the synthesis of gut-derived 5-HT, balancing the transcription factors FOXO1, ATF4, and CREB, and regulating the gut microbiota to inhibit gut-derived 5-HT. These studies provide new directions for the development of novel anti-osteoporosis drugs. Therefore, further research is needed to explore the potential of natural herbal medicine in regulating intestinal-derived 5-HT for the treatment of osteoporosis, as this may bring new and effective treatment methods for osteoporosis patients.

## Author contributions

KS and YiW proposed the framework of this paper. JD and YuW drafted the manuscript. BL and XL integrated the structural information. XZ and XX provided some helpful suggestions in this paper. All authors read and approved the final manuscript.
